# Identification and validation of anoikis-related lncRNAs for prognostic significance and immune microenvironment characterization in ovarian cancer

**DOI:** 10.18632/aging.205439

**Published:** 2024-01-15

**Authors:** Lixue Cao, Shaofen Zhang, Haojie Peng, Yongqing Lin, Zhihui Xi, Wumei Lin, Jialing Guo, Geyan Wu, Fei Yu, Hui Zhang, Haiyan Ye

**Affiliations:** 1 Medical Research Institute, Guangdong Provincial People’s Hospital (Guangdong Academy of Medical Sciences), Southern Medical University, Guangzhou, Guangdong, China; 2 Guangdong Cardiovascular Institute, Guangdong Provincial People’s Hospital, Guangdong Academy of Medical Sciences, Guangzhou, Guangdong, China; 3 Department of Breast Surgery, The Second Affiliated Hospital, Guangzhou Medical University, Guangzhou, Guangdong, China; 4 Department of Gynecology, Guangdong Provincial People’s Hospital, Guangdong Academy of Medical Sciences, Southern Medical University, Guangzhou, Guangdong, China; 5 Biomedicine Research Centre, The Third Affiliated Hospital of Guangzhou Medical University, Guangzhou Medical University, Guangzhou, Guangdong, China; 6 Institute of Human Virology, Key Laboratory of Tropical Disease Control of Ministry of Education, Guangdong Engineering Research Center for Antimicrobial Agent and Immunotechnology, Zhongshan School of Medicine, Sun Yat-sen University, Guangzhou, Guangdong, China

**Keywords:** anoikis, risk model, lncRNA, immune microenvironment, ovarian cancer

## Abstract

Anoikis, a form of apoptotic cell death resulting from inadequate cell-matrix interactions, has been implicated in tumor progression by regulating tumor angiogenesis and metastasis. However, the potential roles of anoikis-related long non-coding RNAs (arlncRNAs) in the tumor microenvironment are not well understood. In this study, five candidate lncRNAs were screened through least absolute shrinkage and selection operator (LASSO), and multivariate Cox regression analysis based on differentially expressed lncRNAs associated with anoikis-related genes (ARGs) from TCGA and GSE40595 datasets. The prognostic accuracy of the risk model was evaluated using Kaplan-Meier survival analysis and receiver operating characteristic (ROC) curves. Furthermore, Kyoto Encyclopedia of Genes and Genomes (KEGG) and gene set enrichment analysis (GSEA) analyses revealed significant differences in immune-related hallmarks and signal transduction pathways between the high-risk and low-risk groups. Additionally, immune infiltrate analysis showed significant differences in the distribution of macrophages M2, follicular T helper cells, plasma cells, and neutrophils between the two risk groups. Lastly, silencing the expression of PRR34_AS1 and SPAG5_AS1 significantly increased anoikis-induced cell death in ovarian cancer cells. In conclusion, our study constructed a risk model that can predict clinicopathological features, tumor microenvironment characteristics, and prognosis of ovarian cancer patients. The immune-related pathways identified in this study may offer new treatment strategies for ovarian cancer.

## INTRODUCTION

Anoikis is a specialized form of programmed cell death triggered by detachment from the extracellular matrix (ECM) [1]. Anoikis resistance has been shown to play a significant role in various cancers, including lung cancer, Ewing sarcoma, prostate cancer, and ovarian cancer [2–6]. Ovarian cancer is the leading cause of death among malignant gynecological cancers, and the majority of patients with advanced-stage disease (stage III or IV) present with malignant ascites [7]. Tumor cells in ascites exhibit anchorage-independent survival, contributing to the metastasis and recurrence of ovarian cancer [8]. Therefore, a comprehensive analysis to identify key drivers of anoikis in ovarian cancer is crucial.

During the process of metastasis, tumor cells with anoikis resistance can survive in the tumor microenvironment (TME), which is heavily influenced by the ECM [6]. Emerging evidence suggests that cancer cells shape the ECM, creating an immune-suppressive microenvironment that reduces the efficacy of immunotherapies [9, 10]. The TME encompasses the complex relationship between tumor occurrence, growth, and metastasis, and the internal and external environment of tumor cells. It consists of various cellular and non-cellular components, including tumor cells, immune cells, extracellular matrix, cytokines, chemokines, and more [11]. It has been demonstrated that ECM components, such as collagens, confer anoikis resistance through B-cell lymphoma (BCL) family proteins [9]. Collagen density and tissue stiffness play a vital role in regulating the infiltration of immune cells. For instance, a high-density matrix leads to a higher ratio of CD4 to CD8 cells, suppressing the activity of cytotoxic T cells in the tumor microenvironment [12]. Moreover, ECM stiffness hampers T-cell migration, while reduction of collagenase reduces stiffness and improves the situation [13]. Therefore, further exploration of the relationship between immune suppression and anoikis resistance in cancer is warranted, as it may provide potential therapeutic targets for immunotherapy.

Multiple evidence proved that long non-coding RNAs (lncRNAs) promote metastasis via regulating anoikis-resistance, which leads to the poor progression of cancer patients [14–16]. LncRNAs have been defined as non-coding RNAs longer than 200 nucleotides, which do not possess the capacity of coding proteins and involving in post-transcriptional regulation of genes, stability of RNA, processing RNA, etc., [17]. For example, silencing the expression of lncRNA APOC1P1-3 decreases anoikis resistance by sponging miRNA-188-3p, thereby blocking the inhibition of Bcl-2 [14]. Additionally, AKT-induced lncRNA VAL promotes anoikis resistance by binding to vimentin and decreasing Trim16-dependent vimentin degradation [15]. Moreover, dysregulation of lncRNAs is involved in the TME and is associated with immune cell infiltration and the response of cancer cells to immunotherapy [18]. In ovarian cancer, lncRNA HOTTIP upregulates the expression of PD-L1 in neutrophils via IL-6 secretion, inhibiting T-cell immunity and contributing to immune evasion by cancer cells [19]. However, the mechanisms by which arlncRNAs regulate the TME in ovarian cancer remain unclear.

In this study, we constructed a novel prognostic signature comprising five arlncRNAs to predict the prognosis of ovarian cancer. Furthermore, we conducted GSEA and immune infiltration analysis to elucidate the regulatory mechanisms of these lncRNAs in ovarian cancer. Additionally, we validated the expression and function of two lncRNAs in ovarian cancer cells to understand their role in regulating anoikis resistance *in vitro*. Our findings provide potential prognostic biomarkers for ovarian cancer and are essential for the development of immunotherapy strategies.

## MATERIALS AND METHODS

### Patients and datasets

The gene expression RNA sequencing (RNA-seq) data of 421 ovarian cancer samples were obtained from The Cancer Genome Atlas (TCGA) database (https://cancergenome.nih.gov) and included complete clinicopathological and survival data. Since the TCGA database lacks normal ovarian tissue data, 32 ovarian cancer samples and 6 normal ovarian samples from the Gene Expression Omnibus (GEO) database were obtained (https://www.ncbi.nlm.nih.gov/geo/). Clinical information for ovarian cancer patients from TCGA is available in Supplementary Table 1, while GSE40595 lacks corresponding clinical information.

### Identification of anoikis-related lncRNAs

Differential expression analysis was performed using the “limma” package (v3.46.0) in R to identify genes differentially expressed between ovarian cancer samples and normal ovarian samples, using the criteria of fold change (|Fc|) > 1 and *p* < 0.01. A total of 434 ARGs with detailed clinical information and prognostic data from the published literature were retrieved [20]. Pearson correlation analysis was conducted to identify lncRNAs correlated with anoikis in ovarian cancer, using a cutoff value of |r| > 0.4 and *p* < 0.001. Kaplan-Meier analysis was performed using the “survival” package (v3.2.7) in R to screen arlncRNAs relevant to the prognosis of ovarian cancer.

### Construction of lncRNA signature model

Univariate Cox regression analysis was performed to assess the prognostic value of each preliminarily screened lncRNA. Multivariate Cox regression and LASSO Cox regression were employed using the “glmnet” package (v4.1.6) in R to identify five characteristic arlncRNAs. A risk model was constructed using these arlncRNAs to predict the prognosis of ovarian cancer. The ovarian cancer patients were divided into high-risk and low-risk groups based on the median risk score calculated using the following formula: Σ (Exp(lncRNA) × Coef(lncRNA)). “Exp” represents the expression level of the lncRNA, and “Coef” represents the coefficient of the corresponding lncRNA. The TCGA samples were randomly divided into two cohorts using the “rsample” package (v1.1.1) in R. The differences in overall survival (OS) between the high-risk and low-risk groups were compared using the “survival” package in R, and Kaplan-Meier survival curves were plotted. The predictive power of the model was validated using ROC curves and the area under the curve (AUC) for 1, 3, and 5 years, using the R package “timeROC.”

### Construction of nomogram

Based on the signature model, a nomogram was established using the R package “rms” (v6.5.0) to predict the 1-, 3-, and 5-year OS of ovarian cancer patients. The nomogram included age, stage, and risk score as variables. Total points were calculated according to the corresponding score of age, stage, and risk score in the nomogram to predict the survival rate at 1, 3, and 5 years. Calibration curves were drawn using the “rms” package in R to assess the predictive accuracy of the nomogram.

### Gene set enrichment analysis

Each ovarian cancer patient was assigned a risk score calculated using the aforementioned formula. The patients were stratified into high-risk and low-risk groups based on the median risk score. GSEA was conducted using the R package “clusterProfiler” (v3.18.1) with hallmark gene sets and KEGG pathways to explore the potential molecular mechanisms promoting ovarian cancer.

### Immune infiltrate analysis

CIBERSORTX (https://cibersortx.stanford.edu/) was used to characterize the proportions of infiltrating immune cells in the different risk groups. The proportions of 22 different immune cell types were evaluated separately in the high-risk and low-risk groups using CIBERSORTX. Genes in the high-risk and low-risk groups were analyzed, and a volcano plot was generated using the “ggplot2” package (v3.4.2) in R, with the criteria of |log2Foldchange| > 1 and *p* < 0.05.

### Cell culture

Caov-3, OVCAR3, and SKOV3 cell lines were obtained from the American Type Culture Collection (ATCC, Manassas, VA, USA). HOSEpiC cells were obtained from ScienCell Research Laboratories (Carlsbad, CA, USA), and HO-8910PM cells were obtained from the Shanghai Cell Library of the Chinese Academy of Sciences (Shanghai, China). All cells were cultured according to the manufacturer’s instructions in a humidified incubator at 37°C with 5% CO2, using culture media supplemented with 10% fetal calf serum and 1% penicillin-streptomycin.

### Reverse transcription and quantitative real-time PCR (qRT-PCR)

Total RNA was extracted from HOSEpiC, Caov-3, HO-8910PM, OVCAR3, and SKOV3 cells using the EZ-press RNA Purification Kit (EZBioscience; Roseville, MN, USA) according to the manufacturer’s protocol. RNA quantification was performed using a microspectrophotometer (KAIAO; Beijing, China). cDNA was synthesized by reverse transcription of RNA using the HiScript III RT SuperMix (Vazyme; Nanjing, China). Glyceraldehyde 3-phosphate dehydrogenase (GAPDH) was used as an internal reference gene. qRT-PCR was performed using the C1000 Touch™ Thermal Cycler (Bio-Rad; Hercules, CA, USA) with ChamQ Universal SYBR qPCR Master Mix (Vazyme; Nanjing, China) The specific primer sequences required for qRT-PCR are provided in Supplementary Table 2. The relative RNA expression was calculated using the 2^−ΔΔCt^ method.

### Cell transfection

Small interfering RNAs (siRNAs) targeting PRR34_AS1 or SPAG5_AS1 were purchased from Tsingke (Guangzhou, China). A total of 20 nM siPRR34_AS1 and siSPAG5_AS1 were transfected into SKOV3 and OVCAR3 cells using Lipofectamine RNAiMAX (Invitrogen, Carlsbad, CA, USA) Reagent according to the manufacturer’s instructions. Cells were collected after 48 hours for quantitative real-time PCR to measure the knockdown efficiency and other experiments. The siRNA sequences are provided in Supplementary Table 2.

### Flow cytometry

Cells were seeded in six-well plates after transfection at a density of 1 × 10^5^ cells per well. After 48 hours of incubation, the apoptosis rate of cells was measured using the Annexin V-FITC Apoptosis Detection Kit (KeyGEN, Nanjing, China). Cells were washed twice with PBS, centrifuged at 3000 rpm for 5 minutes, resuspended in 500 μL of binding buffer, and stained with 5 μL of Annexin V-FITC and 5 μL of Propidium Iodide (PI) in the dark for 15 minutes at room temperature. The apoptosis rate of the cells was measured using a Beckman Coulter Flow Cytometry (Beckman, Krefeld, Germany).

### Anoikis assay

A total of 1 × 10^5^ cells were seeded in ultra-low-attachment 96-well plates and incubated for 24 hours. The Calcein/PI Assay Kit (Beyotime, Shanghai, China) was used to analyze the levels of living and dead cells. The plate was centrifuged at 400 × g for 5 minutes, and the cells were washed once with PBS. Cells were stained with 100 μL of Calcein AM/PI (1:1) in the dark for 30 minutes at 37°C. Cell immunofluorescence was performed using the Electronic Ballast EBQ 100–04 (Leistungselektronik JENA GmbH, Jena, Germany). Living cells appeared green, while dead cells appeared red in the fluorescent images.

### Statistical analysis

All statistical analyses were conducted using R version in RStudio and GraphPad Prism 9.0 software. Graphs were produced using the “ggplot2” package in RStudio. Kaplan-Meier survival analysis and ROC curves were used to assess the predictive value of the signature model in different groups. Univariate, multivariate, and LASSO Cox regression analyses were performed to screen for anoikis-related lncRNAs. Pearson correlation analysis was conducted to explore the correlation between different immune cells and ovarian cancer. Statistical significance was defined as a *p*-value < 0.05.

### Availability of data and materials

Data are available from the corresponding author upon reasonable request.

## RESULTS

### Screening of prognostic anoikis-related differentially expressed lncRNAs in ovarian cancer

**Figure 1:**
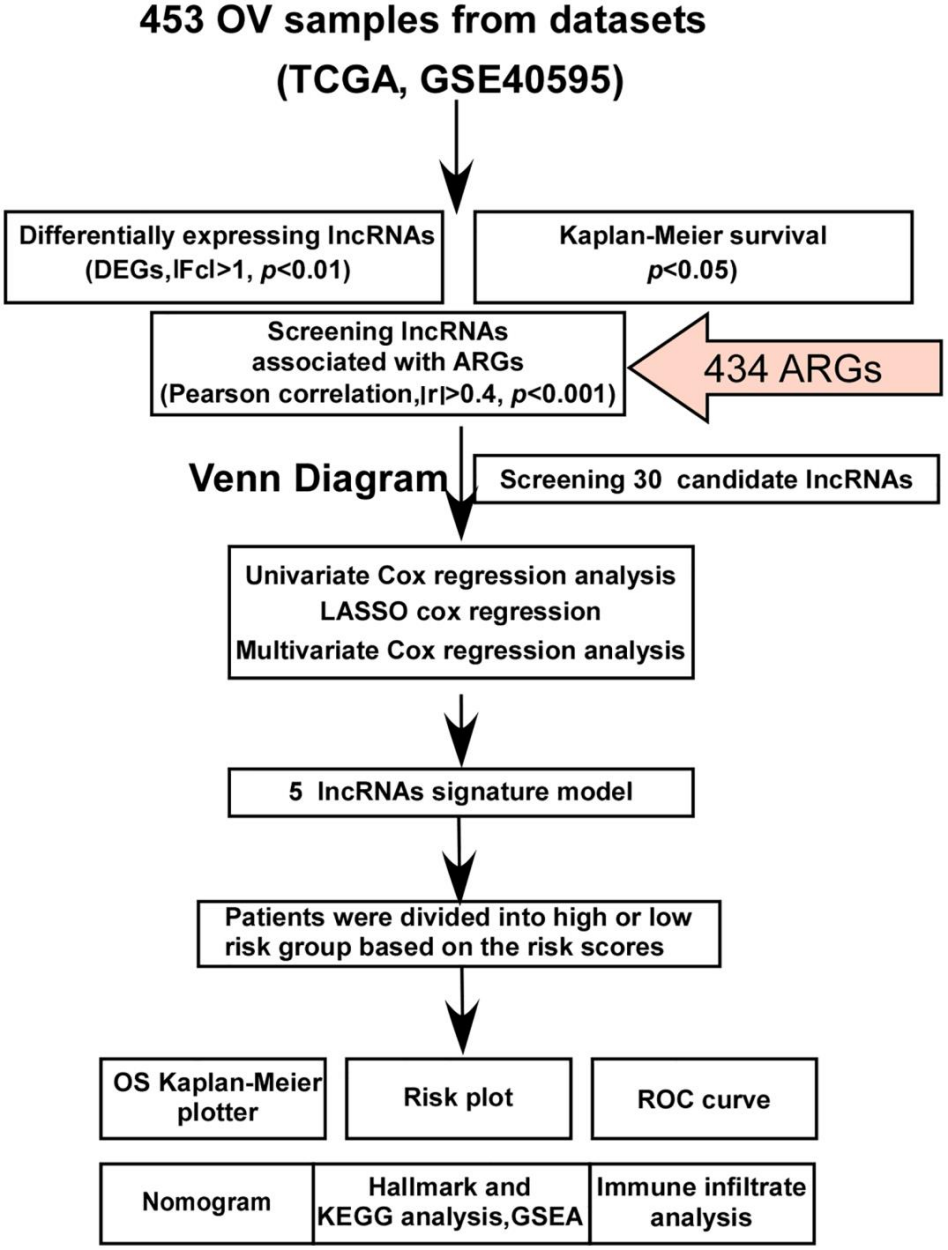
**Flowchart of the study.** Thirty candidate lncRNAs were identified through Venn diagram analysis of differentially expressed lncRNAs, Kaplan-Meier survival analysis, and lncRNAs associated with ARGs (anoikis-related genes). Subsequently, a novel cancer signature model consisting of five prognostic arlncRNAs was developed for ovarian cancer patients using univariate Cox regression, LASSO analysis, and multivariate Cox regression analysis. The five arlncRNAs signature model was established and the patients were divided into two risk groups based on the risk scores. The accuracy and potential function of this signature were assessed through various analyses, including OS (overall survival) Kaplan-Meier analysis, risk plot analysis, ROC (receiver operating characteristic) curve analysis, nomogram construction, hallmark analysis, KEGG (Kyoto Encyclopedia of Genes and Genomes) analysis, GSEA (gene set enrichment analysis), and immune infiltrate analysis. OV refers to ovarian cancer, TCGA refers to The Cancer Genome Atlas, lncRNAs stands for long noncoding RNAs, DEGs represents differentially expressed genes, ARGs denotes anoikis-related genes, LASSO refers to least absolute shrinkage and selection operator, OS refers to overall survival, ROC stands for receiver operating characteristic, KEGG refers to Kyoto Encyclopedia of Genes and Genomes, and GSEA represents gene set enrichment analysis.

**Figure 2:**
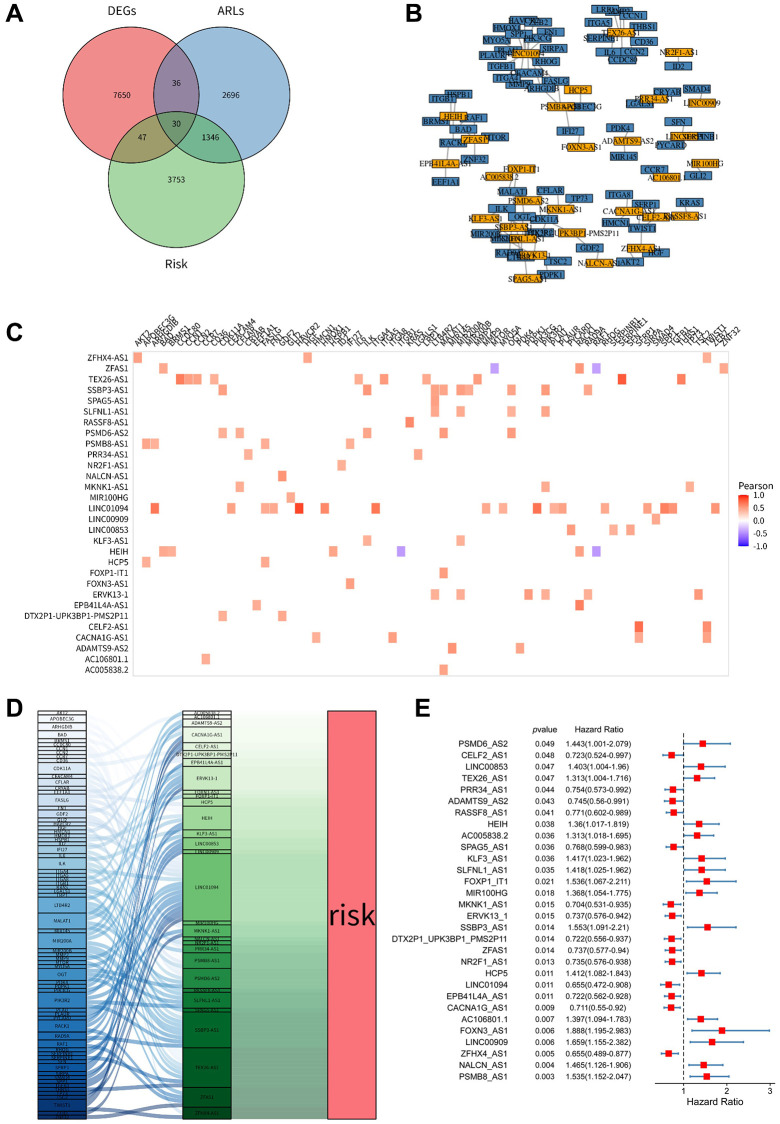
**Identification of anoikis-related lncRNAs in ovarian cancer (OV).** (**A**) Venn diagram showing the 30 lncRNAs identified through the intersection of differentially expressed lncRNAs, Kaplan-Meier survival analysis, and lncRNAs associated with ARGs. (**B**) Coexpression network depicting the relationship between the 30 differentially expressed lncRNAs, DEGs (differentially expressed genes), and ARLs (anoikis-related lncRNAs) based on Pearson's correlation coefficient (R > 0.4, *p* < 0.001). (**C**) Correlation heatmap illustrating the correlation between the 30 lncRNAs and ARGs. The color intensity represents the strength of the correlation. (**D**) Sankey diagram demonstrating the connections between the 30 lncRNAs and ARGs. (**E**) Forest plots displaying the results of univariate Cox regression analysis for the 30 lncRNAs. OV refers to ovarian cancer, DEGs stands for differentially expressed genes, ARGs denotes anoikis-related genes, and ARLs represents anoikis-related lncRNAs.

The research flow diagram is shown in Figure 1. We downloaded gene expression RNA-seq data of 421 ovarian cancer samples from the TCGA database. Additionally, microarray data of 32 ovarian cancer samples and 6 normal ovarian cell samples were obtained from the GEO database (GSE40595). Using differential expression analysis with the criteria of |Fc| > 1 and *p* < 0.01, we identified 7763 differentially expressed lncRNAs between ovarian cancer and normal ovarian cells. To identify lncRNAs involved in the process of anoikis resistance, we obtained 434 ARGs from published literature. By performing Pearson correlation analysis (|r| > 0.4, *p* < 0.001), we identified 4108 arlncRNAs. Taking the intersection of differentially expressed lncRNAs (DEGs) and arlncRNAs, we obtained 66 candidate lncRNAs. Kaplan-Meier survival analysis revealed 5176 prognostic lncRNAs in ovarian cancer based on *p* < 0.05. Using a Venn diagram analysis, we identified 30 candidates prognostic arlncRNAs (Figure 2A). We constructed a lncRNA-gene coexpression network (Figure 2B) and visualized the degree of correlation between the candidate arlncRNAs and ARGs (Figure 2C). The Sankey diagram displayed the links between the candidate lncRNAs and ARGs (Figure 2D). Univariate Cox regression analysis revealed that the 30 candidate arlncRNAs were significantly associated with the prognostic value of ovarian cancer patients, as shown in the forest plot (Figure 2E).

### Construction and validation of anoikis-related lncRNAs

**Figure 3:**
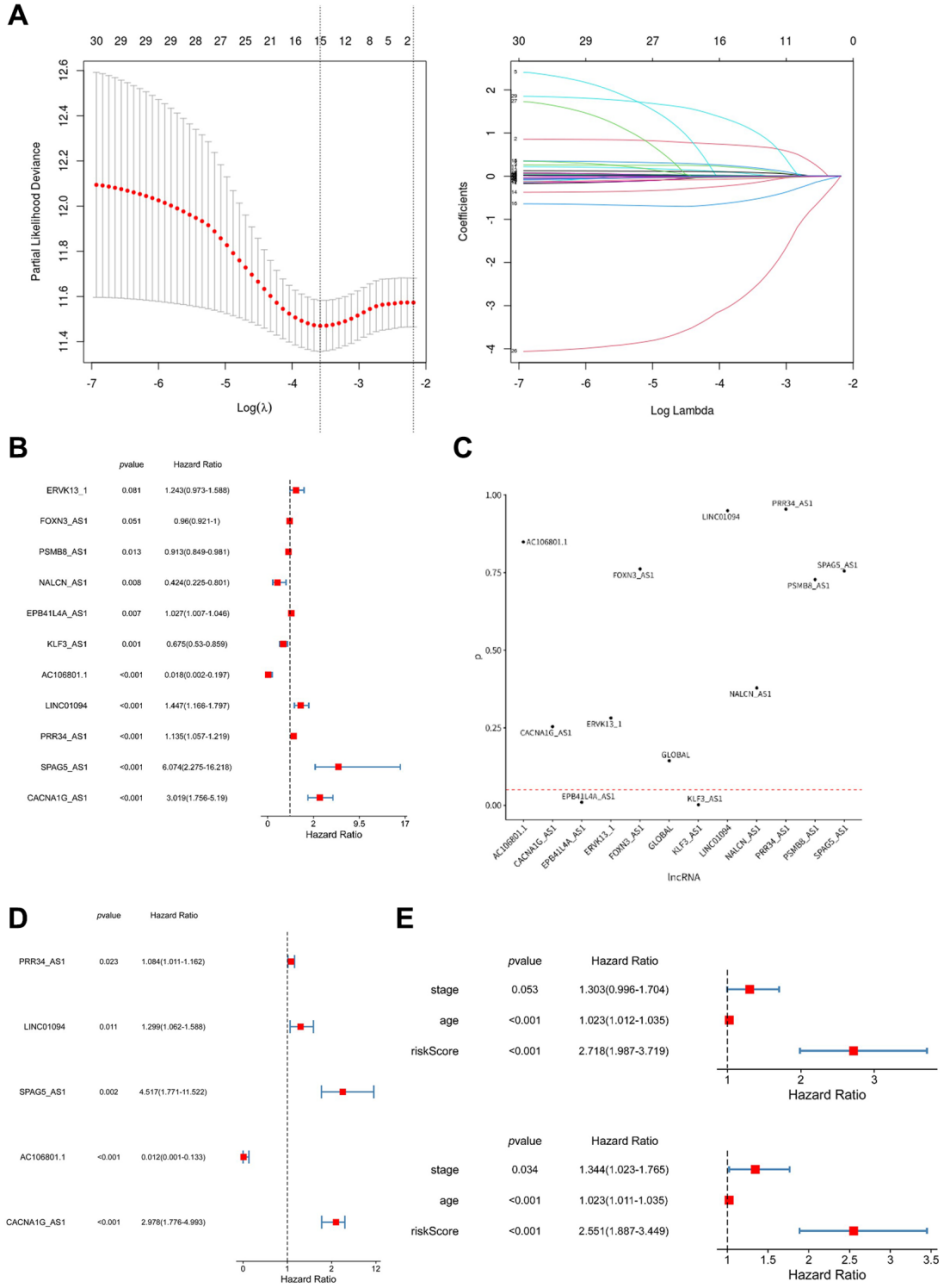
**Construction of a prognostic model consisting of five anoikis-related lncRNAs.** (**A**) LASSO regression analysis and partial likelihood deviance were performed to identify the prognostic lncRNAs. (**B**) Four lncRNAs were excluded based on the multivariate Cox regression analysis. (**C**) Two lncRNAs were excluded based on the PH assumption (proportional hazards assumption). (**D**) Five arlncRNAs were finally identified by performing multivariate Cox regression analysis. (**E**) Univariate and multivariate Cox regression analysis was conducted to determine the independent risk factors.

**Figure 4:**
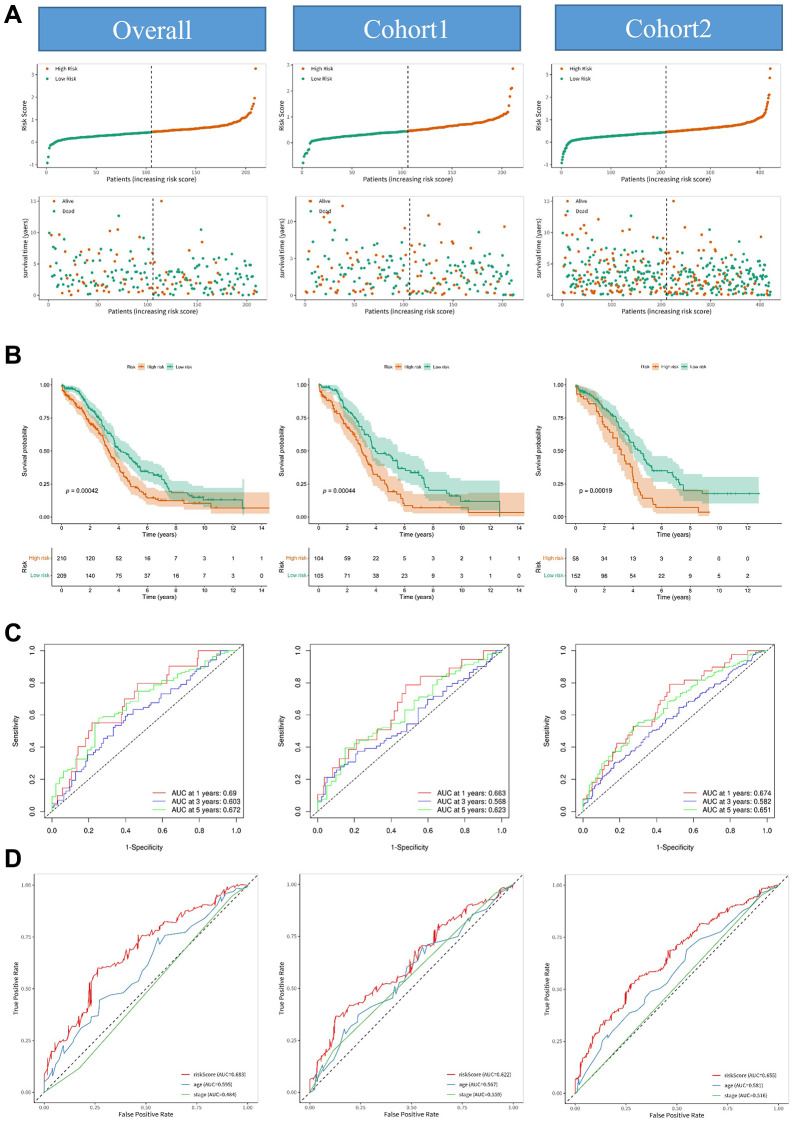
**Construction and validation of the prognostic model consisting of anoikis-related lncRNAs.** (**A**) Distribution diagrams showing the risk scores and survival status of the overall cohort, cohort 1, and cohort 2. (**B**) Kaplan-Meier curves illustrating significant differences in the overall survival rate between the high-risk group and low-risk group in the overall cohort, cohort 1, and cohort 2. (**C**) ROC curves depicting the predictive performance of the risk model for 1-year, 3-year, and 5-year overall survival in the overall cohort, cohort 1, and cohort 2. (**D**) ROC curves comparing the AUC (area under the curve) values of the risk score, age, and stage in the overall cohort, cohort 1, and cohort 2.

To further validate the prognostic potential of arlncRNAs, we conducted LASSO Cox regression analysis, identifying 15 arlncRNAs (Figure 3A). Subsequently, four arlncRNAs were eliminated through multivariate Cox regression analysis (Figure 3B). Following the proportional hazards assumption, we retained nine arlncRNAs (Figure 3C). The final step involved performing multivariate Cox regression analysis, resulting in the identification of five arlncRNAs with significant prognostic value (Figure 3D). Ultimately, we identified 5 prognostic arlncRNAs as a novel cancer signature model for ovarian cancer patients. The risk score in this model was calculated using the following formula: Risk score = (LINC01094 × 0.26153966441116) + (AC106801.1 × −4.44168323698003) + (PRR34_AS1 × 0.0806604036265822) + (SPAG5_AS1 × 1.50780023844841) + (CACNA1G_AS1 × 1.09115566233272). Univariate and multivariate Cox regression analysis revealed that age and the risk score of the 5 arlncRNAs were independent prognostic factors in ovarian cancer. The Hazard Ratio (HR) of age was 1.023 (95% CI: 1.012–1.035), while the HR of the risk score was 2.781 (95% CI: 1.987–3.719) (Figure 3E). The risk score performed better than age in terms of prognostic value. We established a nomogram to predict one-, three-, and five-year overall survival for ovarian cancer patients by incorporating clinicopathological features and the risk score (Supplementary Figure 1A). Calibration curves were employed to assess the alignment between observed overall survival and predicted ovarian cancer survival. The results indicate that the prediction of overall survival was accurate in capturing the dynamics of ovarian cancer survival (Supplementary Figure 1B). We further evaluated the prognostic value of the 5 arlncRNAs signature model in various aspects. Ovarian cancer samples from TCGA were randomly divided into two cohorts, and the ovarian cancer samples in each cohort were stratified into high-risk and low-risk groups based on the median score calculated using the risk score formula (Figure 4A). As shown in Figure 4B, ovarian cancer patients in the high-risk group had worse prognosis, while the low-risk group had a higher survival rate. The accuracy of this model was validated using ROC curves, with AUC values of 0.69, 0.603, and 0.672 for one, three, and five years, respectively, indicating superior predictive value of the prognostic signature model (Figure 4C). ROC curves were also used to compare the performance of the risk score with stage and age, demonstrating that the risk score performed better (Figure 4D). Therefore, the risk score based on the 5 prognostic arlncRNAs accurately predicted the survival of ovarian cancer patients.

### Relationship between the 5 arlncRNAs signature and clinicopathological parameters in ovarian cancer patients

**Figure 5:**
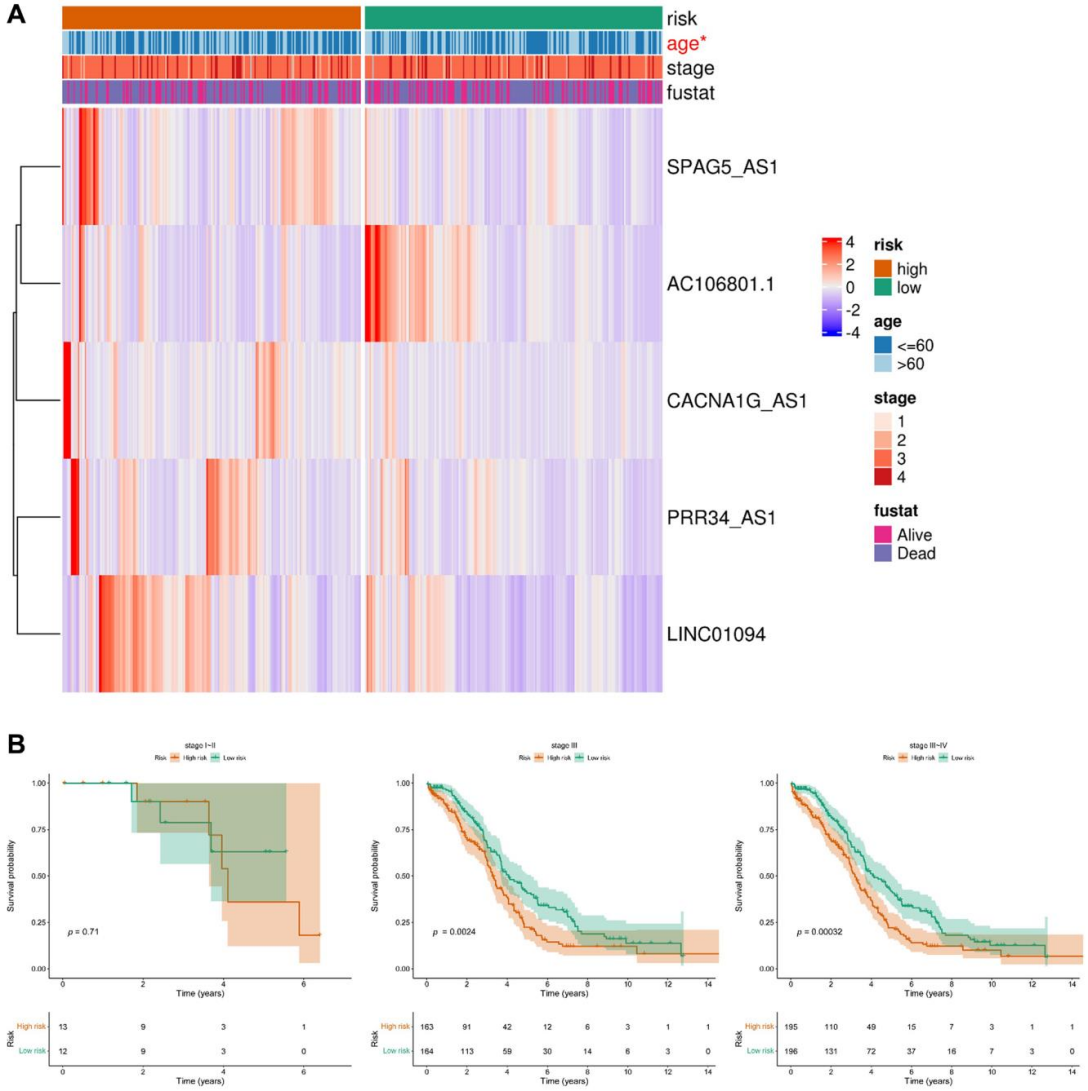
**Relationship between the prognostic model consisting of five anoikis-related lncRNAs and clinicopathological features in ovarian cancer patients.** (**A**) Heatmap displaying the distribution of the expression levels of the five lncRNAs in the high-risk group and low-risk group based on different clinicopathological features. (**B**) Kaplan-Meier curves illustrating the overall survival in different stages.

Among the five lncRNAs, four were considered risk lncRNAs, and they were upregulated in the high-risk group in the ovarian cancer database, except for AC106801.1, which was a protective marker (Figure 5A). To explore independent factors in the signature model, we compared different clinicopathological parameters between the high-risk and low-risk groups. The heatmap displayed the correlation of age, stage, and fustat in the high-risk and low-risk groups (Figure 5A). Interestingly, we found a significant difference in age between the high-risk and low-risk groups (*p* < 0.05). Kaplan-Meier survival analysis showed that ovarian cancer patients in the low-risk group had a higher survival rate than those in the high-risk group at stage III and stage III-IV (Figure 5B), while there was no significant difference observed in early-stage ovarian cancer patients. Due to the non-specific symptoms contributing to over 75% of ovarian cancer diagnoses at an advanced stage [21], the clinical study’s inclusion of early-stage ovarian cancer patients is restricted. The limited sample size in the early-stage group may hinder the adequate detection of survival rate differences between low-risk and high-risk stages I-II, necessitating further examination. Therefore, these results indicated that the five arlncRNAs had an excellent ability to predict prognosis in ovarian cancer patients, especially at late stages (III/IV).

### Discovery of molecular functions and pathways of the 5 arlncRNAs through KEGG and GSEA enrichment analysis

**Figure 6:**
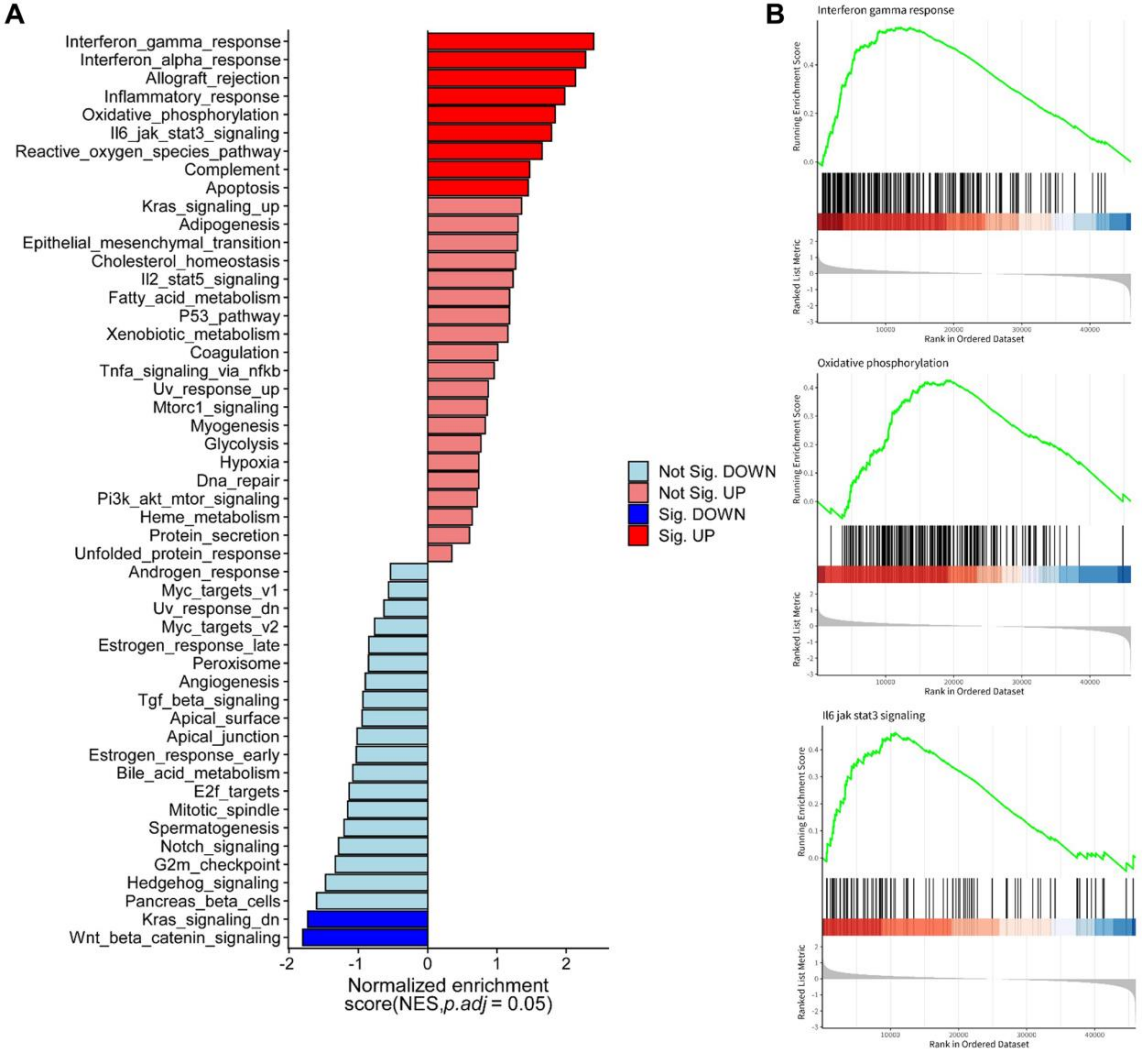
**Molecular functions and pathways associated with the five anoikis-related lncRNAs identified through hallmark and GSEA enrichment analysis.** (**A**) Pathway enrichment analysis using hallmark databases. (**B**) GSEA analysis revealing enriched pathways in the high-risk group and low-risk group.

To further explore the underlying biological functions and mechanisms associated with the risk groups defined by the 5 arlncRNAs signature, we performed KEGG and GSEA enrichment analyses. The enrichment plot visualized the results of Hallmark and KEGG pathway enrichment analyses (Figure 6A and Supplementary Figure 2A), revealing that oxidative phosphorylation (OXPHOS) and immune-related pathways were significantly enriched in the high-risk group. GSEA analysis further demonstrated enrichment of the interferon gamma response (IFN-γ), OXPHOS, IL-6/JAK/STAT3 signaling pathway, chemical carcinogenesis-reactive oxygen species (ROS), chemokine signaling pathway, and cytokine-cytokine receptor interaction in the high-risk group, indicating their involvement in promoting ovarian cancer (Figure 6B and Supplementary Figure 2B). In summary, these results suggested that the risk score of the 5 arlncRNAs signature predominantly predicts prognostic survival through immune-related pathways and OXPHOS in ovarian cancer.

### Immune infiltrate analysis of the signature model

**Figure 7:**
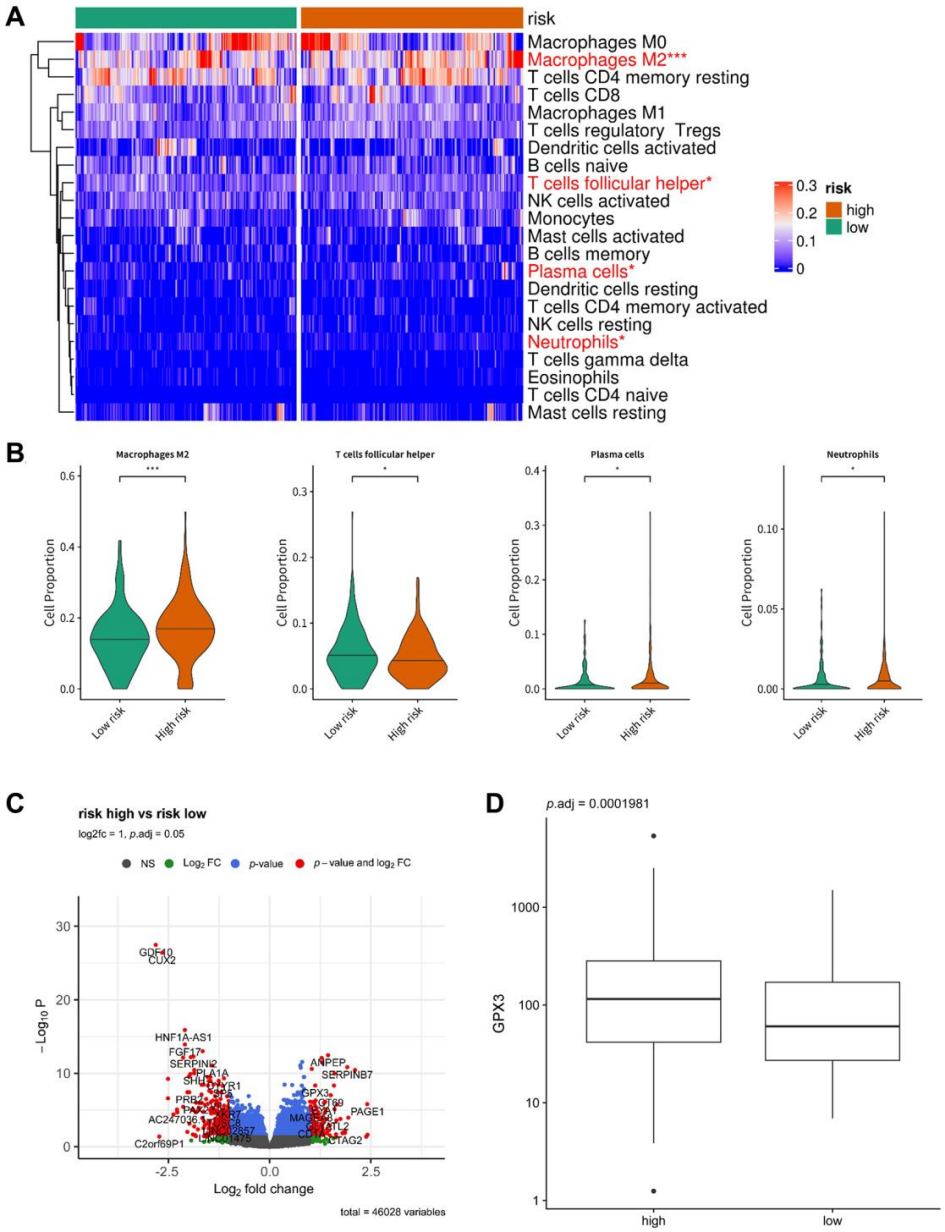
**Immune infiltrate analysis of the prognostic model.** (**A**) Heatmap displaying the distribution of 22 different immune cell types in the high-risk group and low-risk group. (**B**) Box plots illustrating the proportion of macrophages M2, T cells follicular helper, plasma cells, and neutrophils in the low-risk group and high-risk group. (**C**) Volcano plot depicting the differentially expressed genes between the low-risk group and high-risk group (*p* < 0.05, |log2Foldchange| > 1). (**D**) Box plot showing the expression of GPX3 in the low-risk group and high-risk group.

To investigate the relationship between the 5 arlncRNAs signature and the immune system, we compared the proportions of 22 different immune cells in the high-risk and low-risk groups using the CIBERSORTX algorithm, and a heatmap was generated (Figure 7A). The distribution of macrophages M2, T cells follicular helper, plasma cells, and neutrophils significantly differed between the two risk groups. Box plots showed that the proportion of T cells follicular helper was higher in the low-risk group, while the proportion of macrophages M2, plasma cells, and neutrophils was higher in the high-risk group (Figure 7B). To identify potential targets in ovarian cancer, we analyzed differentially expressed genes between the high-risk and low-risk groups (*p* < 0.05, |log2Foldchange| > 1) and visualized them with a volcano plot (Figure 7C). A box plot showed that the expression of glutathione peroxidase 3 (GPX3) was significantly higher in the high-risk group (Figure 7D). GPX3 is an extracellular antioxidant enzyme and the main ROS scavenger in plasma [22]. It is highly expressed in ovarian cancer and associated with platinum resistance and survival in ascites by protecting cancer cells from extracellular oxidative stressors [23]. Thus, the 5 arlncRNA signature regulates immune infiltration and oxidative stress in ovarian cancer.

### Validation of the expression and function of the five anoikis-related lncRNAs in ovarian cancer cell lines

**Figure 8:**
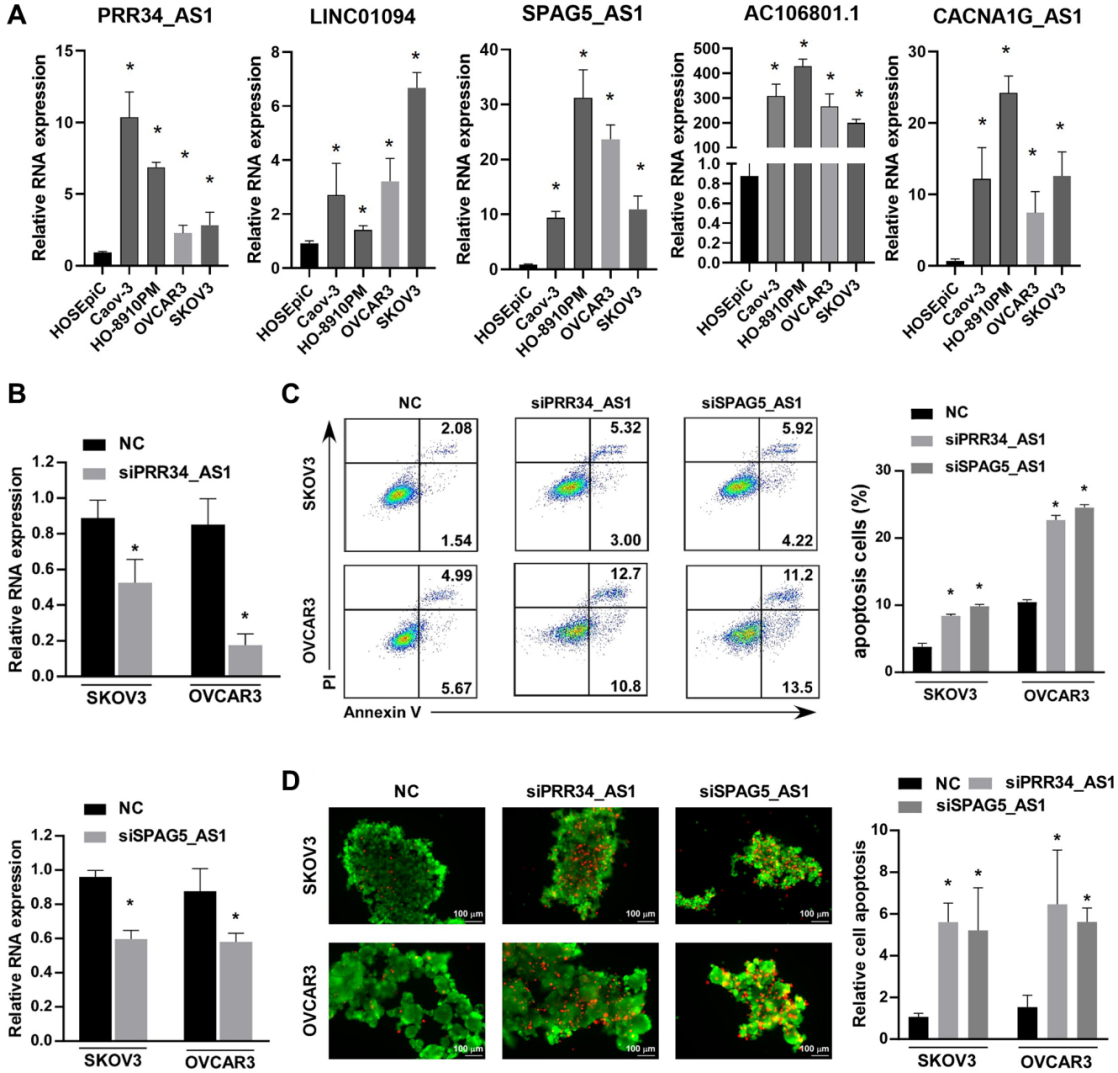
**Validation of the expression and function of the five anoikis-related lncRNAs in ovarian cancer cell lines.** (**A**) RT-PCR detection of the expression levels of PRR34_AS1, LINC01094, SPAG5_AS1, AC106801.1, and CACNA1G_AS1 lncRNAs in various ovarian cancer cell lines (Caov3, HO-8910PM, OVCAR3, SKOV3) and normal ovarian cells (HOSEpiC). (**B**) Validation of the relative RNA expression in SKOV3 and OVCAR3 cells after knockdown of PRR34_AS1 or SPAG5_AS1. (**C**) Flow cytometry analysis of the proportion of apoptotic cells in SKOV3 and OVCAR3 cells after knockdown of PRR34_AS1 and SPAG5_AS1, respectively. (**D**) Fluorescent image showing live/dead SKOV3 and OVCAR3 cells after knockdown of PRR34_AS1 or SPAG5_AS1 using calcein-M and PI staining (living cells in green fluorescence, dead cells in red fluorescence).

To further validate the function of the 5 arlncRNAs in ovarian cancer, the expression and apoptosis assays were performed. qRT-PCR analysis revealed significantly higher expression levels of PRR34_AS1, LINC01094, SPAG5_AS1, AC106801.1, and CACNA1G_AS1 in ovarian cancer cell lines (Caov-3, HO-8910PM, OVCAR3, and SKOV3) compared to HOSEpiC, a cell line of normal ovarian cells (*p* < 0.05) (Figure 8A). However, the inconsistent levels of AC106801.1 in ovarian cancer cell lines, which possibly due to the limitations of cell line experiments, prompt us to further verify in clinical specimens. This preliminary inference suggested that these arlncRNAs may promote the development of ovarian cancer. To investigate the specific effects of these lncRNAs, PRR34_AS1 or SPAG5_AS1 was silenced using siRNA in SKOV3 and OVCAR3 cells. qRT-PCR confirmed the downregulation of PRR34_AS1 and SPAG5_AS1 expression in SKOV3 and OVCAR3 cells treated with siRNA (Figure 8B). Apoptosis assays using PI and Annexin V staining, followed by flow cytometry analysis, showed a significant increase in the proportion of apoptotic cells in SKOV3 and OVCAR3 cells upon silencing PRR34_AS1 or SPAG5_AS1 (Figure 8C). Furthermore, to assess whether these arlncRNAs are involved in anoikis resistance, SKOV3 and OVCAR3 cells with silenced PRR34_AS1 or SPAG5_AS1 were plated into ultra-low attachment plates, and the anoikis cells were detected using a calcein/PI assay. The fluorescent images showed a significant increase in apoptotic cells (red fluorescence) in SKOV3 and OVCAR3 cells transfected with siPRR34_AS1 or siSPAG5_AS1 (Figure 8D). These findings verified that silencing the expression of PRR34_AS1 or SPAG5_AS1 induces anoikis in ovarian cancer cells.

## DISCUSSION

An increasing number of studies have been focusing on understanding the role of anoikis resistance in cancer, as it is considered a crucial factor in cancer metastasis [24, 25]. Ovarian cancer is a malignant gynecological cancer characterized by widespread peritoneal dissemination, which leads to a poor prognosis [26, 27]. The dissemination and attachment of ovarian cancer cells in the peritoneal cavity, particularly in the omental and bowel regions, contribute to anoikis resistance and cancer progression [28]. The peritoneal cavity and the accumulation of peritoneal fluid, known as ascites, consist of diverse cell populations, including macrophages, lymphocytes, and leukocytes, which play a significant role in the progression of ovarian cancer [29, 30]. Therefore, there is an urgent need for a systematic analysis to identify the key drivers of anoikis resistance in ovarian cancer.

However, the combined effect of lncRNAs and anoikis in ovarian cancer remains unknown. In this study, we comprehensively analyzed 30 candidates prognostic lncRNAs related to anoikis from TCGA and GEO (GSE40595), which were correlated with 434 ARGs identified in published literature. Furthermore, we validated five prognostic lncRNAs after a comprehensive analysis, and developed a prognostic signature model to predict the prognosis of ovarian cancer patients. We also constructed a nomogram to predict the 1-, 3-, and 5-year survival rates in ovarian cancer patients, providing a convenient tool for clinical use. The predictive power of the signature model was validated using ROC curves. To gain further insights into the potential mechanisms underlying ovarian cancer progression, we stratified patients into high-risk and low-risk groups based on the risk score model. The patients in the low-risk group demonstrated a higher survival rate compared to those in the high-risk group, particularly at stage III and stage III-IV. LncRNA exhibit resistance to RNase degradation and stability in body fluids [31]. Their higher abundance than protein-coding genes makes lncRNAs advantageous biomarkers [21, 32]. Liquid biopsy advancements enable the detection of lncRNAs in blood or urine, exemplified by urine PCA3 as a prostate cancer diagnostic marker [33]. Targeting lncRNAs with antisense nucleotides (ASO), RNA interference (siRNA), or peptide nucleic acid (PNA) offers therapeutic avenues [34]. Inhibiting lncRNA-SAMMSON and lncRNA-ceruloplasmin demonstrated anti-cancer effects in melanoma and ovarian cancer, respectively [35, 36]. Future detection of the five arlncRNAs in body fluids could predict ovarian cancer prognosis and offer therapeutic targets.

Furthermore, we performed KEGG and GSEA analyses to explore the differences in enriched pathways between the high-risk and low-risk groups, based on the risk scores of the five identified lncRNAs. The results revealed significant differences in immune-related hallmarks and signal transduction pathways between the two groups. We found that the IFN-γ response and the IL-6/JAK/STAT3 signaling pathway were enriched in the high-risk group. Previous studies have extensively investigated the anti-tumor role of IFN-γ in cancer over the past decades. However, it has been found that IFN-γ can upregulate the expression of PD-L1, thereby promoting cancer progression. Moreover, IFN-γ can activate the JAK/STAT pathway, a classic signal transduction pathway involved in cell proliferation, migration, and apoptosis [37]. In ovarian cancer, the activation of STAT3 has been shown to induce anoikis resistance by altering extracellular matrix production, thus promoting cancer progression [38]. Additionally, we observed enrichment of ROS and OXPHOS in the high-risk group. Abnormally elevated levels of ROS have been associated with malignant characteristics such as migration and invasion [39]. Furthermore, ROS production in cancer cells is particularly important for anoikis resistance, and it is dependent on the PI3K/AKT and ERK signaling pathways [40]. While the Warburg effect has traditionally suggested down-regulation of OXPHOS in tumor cells, recent studies have revealed that tumor cells can switch between glycolysis and OXPHOS in different metabolic environments [41, 42]. Moreover, cancer stem cells have been closely linked to oxidative phosphorylation [43–45]. Inhibition of OXPHOS has emerged as a potential therapeutic target in ovarian cancer. Therefore, further exploration of the regulatory mechanisms involving the identified five lncRNAs in ovarian cancer is warranted.

Considering the increasing importance of the tumor microenvironment (TME) and immunotherapy in cancer treatment, including ovarian cancer, we conducted immune infiltrate analysis to investigate the differences between the high-risk and low-risk groups. M2-like macrophages, plasma cells, and neutrophils were found to be enriched in the high-risk group. Numerous studies have suggested that M2-like macrophages promote cancer development by helping cancer cells evade immune clearance [46, 47]. M2 macrophages contribute to tumor development primarily by inhibiting immune clearance, promoting proliferation, and stimulating angiogenesis [48]. TAMs have been shown to contribute to the genetic instability of cancer cells by recruiting ROS [46]. Furthermore, M2-like macrophages can impair the activity of dendritic cells (DCs), natural killer cells (NKs), and cytotoxic T lymphocytes (CTLs) through the release of cytokines [47]. Neutrophils and plasma cells were also observed to be infiltrated in the high-risk group. Neutrophils have been reported to promote ovarian cancer metastasis. Neutrophils, which account for the largest proportion of granulocytes, possess chemotactic, phagocytic, and bactericidal effects. In the TME, neutrophils acquire a suppressor phenotype, leading to impaired signal transduction between neutrophils and T cells, resulting in T cell immunoparalysis and weakened anti-tumor effects [49]. Neutrophils are recruited to the omentum before metastasis in ovarian cancer, promoting the formation of a microenvironment conducive to ovarian cancer cell survival. Furthermore, ovarian cancer cells secrete specific cytokines to recruit neutrophils to the site of metastasis, where they release neutrophil extracellular traps (NETs) that capture isolated ovarian cancer cells and promote metastasis. B cells are classified into six subtypes based on marker expression, including naive B cells, germinal center, IgM memory, switched memory, memory-like, and plasma cells [50]. Plasma cells play an anti-tumor role by producing antibodies. High levels of plasma cells have been associated with better prognosis in patients with gastric cancer, non-small cell lung cancer, and other malignant tumors. However, the effect of plasma cells appears to be opposite in breast cancer and cervical cancer, suggesting that plasma cells may have different effects in different malignancies [51] Therefore, these findings provide insights into the relationship between the five identified lncRNAs and the TME, potentially identifying novel targets for immunotherapy in ovarian cancer.

To further explore potential targets in ovarian cancer, we analyzed the DEGs between the high-risk and low-risk groups and visualized them using a volcano plot (Figure 7C). We observed that GPX3, an extracellular antioxidant, was significantly upregulated in the high-risk group, which supports the extracellular antioxidant defense of ovarian cancer cells and contributes to the progression of ovarian cancer [52]. In addition, GPX3 plays a beneficial role in ovarian cancer cell clonogenicity and survival, which acts as a key measure of anchorage-independent cell survival. By eliminating extracellular oxidants like H_2_O_2_, GPX3 is crucial for ovarian cancer cells to thrive in ascites [23, 52]. Consistently, oxidative stress-related pathways, such as OXPHOS and chemical carcinogenesis-ROS, were enriched in the high-risk group, indicating their involvement in ovarian cancer promotion (Figure 6B and Supplementary Figure 2B). GPX3 plays a critical role in the response to oxidative stress and has been implicated in macrophage escape [53]. Thus, the inhibition of GPX3 in ovarian cancer cells may hold potential for the development of novel anti-tumor drugs in the future.

Additionally, we examined the expression of the five identified lncRNAs in various ovarian cancer cell lines, demonstrating their upregulation in ovarian cancer cells. We also conducted a series of apoptosis-related experiments, validating that the inhibition of PRR34_AS1 or SPAG5_AS1 can promote apoptosis and anoikis in ovarian cancer cell lines. However, our study has several limitations that need to be addressed. First, we lacked validation using clinical samples in our study. Second, the specific mechanisms by which these lncRNAs affect anoikis, inhibit immune infiltration, and promote ovarian cancer progression remain unknown. Further research is warranted to address these limitations and gain a better understanding of the underlying mechanisms.

## CONCLUSIONS

In conclusion, the study conducted a comprehensive analysis of prognostic arlncRNAs in ovarian cancer. It identified a 5 arlncRNA signature that accurately predicted the prognosis of ovarian cancer patients. The signature model demonstrated its potential as an independent prognostic factor and outperformed age and stage in predicting survival. The study also provided insights into the biological functions and pathways associated with the signature, highlighting immune-related pathways and oxidative phosphorylation. Moreover, the 5 arlncRNAs were found to regulate immune infiltration and oxidative stress in ovarian cancer. The expression of these arlncRNAs were validated in ovarian cell lines and the biological function of siPRR34_AS1 or siSPAG5_AS1 were validated to promote apoptosis and anoikis in ovarian cancer cell lines.

## Supplementary Materials

Supplementary Figures

Supplementary Tables
