# Supplementary material for: Identification and validation of anoikis-related lncRNAs for prognostic significance and immune microenvironment characterization in ovarian cancer
Source: Aging (Albany NY). 2024 Jan 15;16(2):1463–83. doi: 10.18632/aging.205439 (PMC10866438; doi:10.18632/aging.205439)
Supplement: Supplementary Figures [file aging-16-205439-s001.pdf]

SUPPLEMENTARY FIGURES

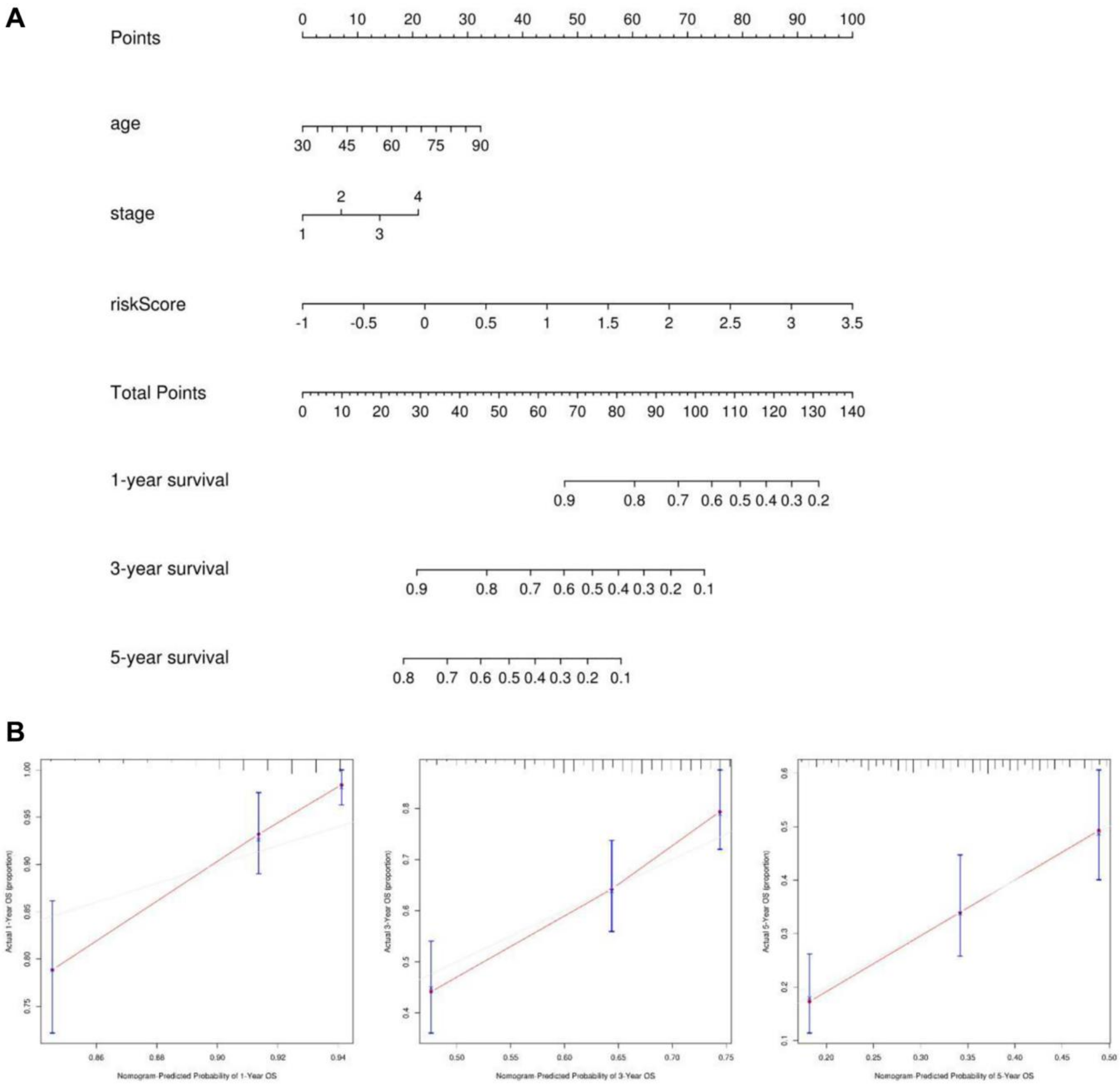

Supplementary Figure 1. Construction of nomogram and validation its predictive value. (**A**) Nomogram was constructed to predict the 1-year, 3-year, and 5-year overall survival of ovarian cancer patients. (**B**) Calibration curves were generated to assess the predictive accuracy of the nomogram for the 1-year, 3-year, and 5-year overall survival rates.

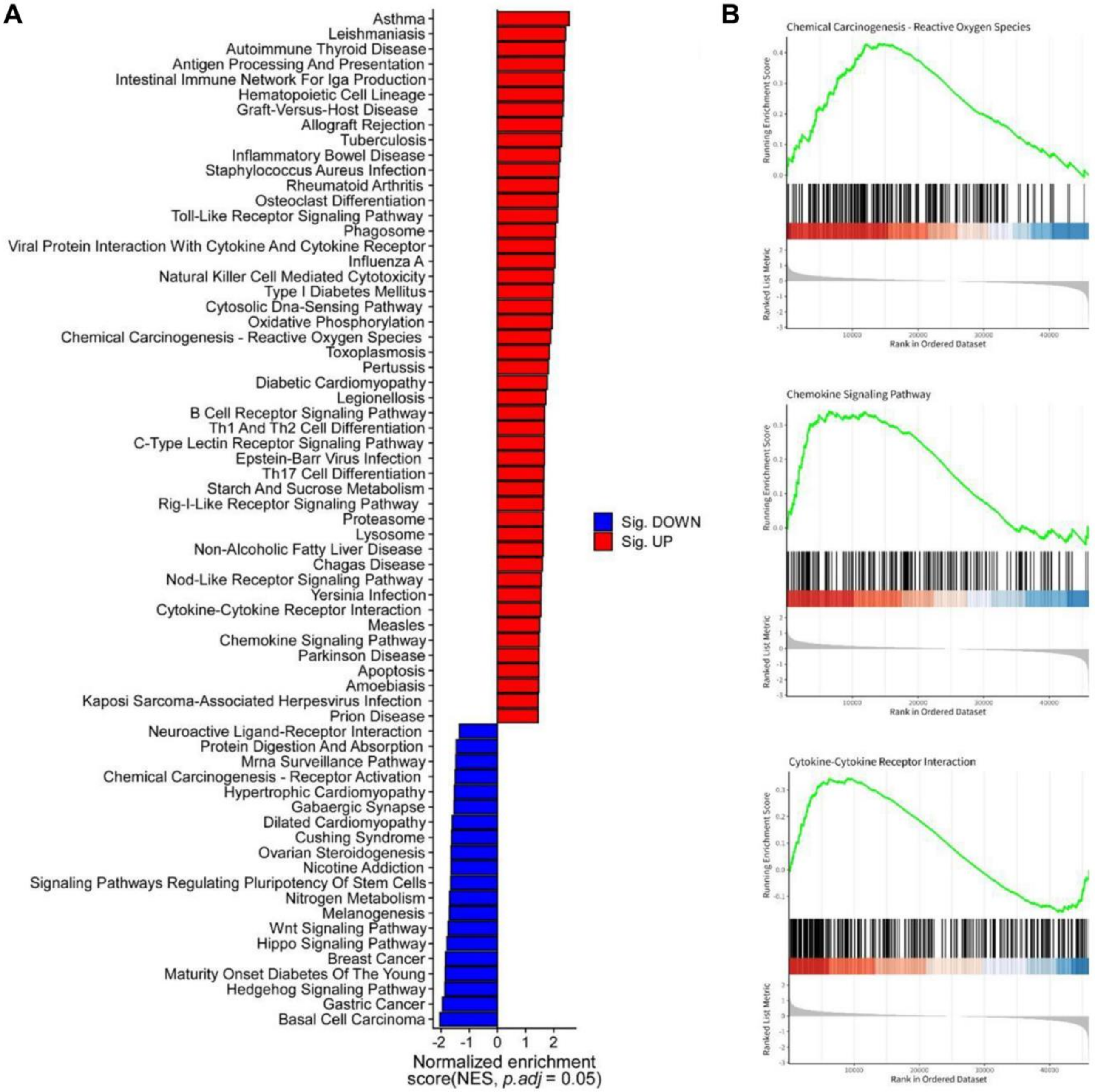

Supplementary Figure 2. Molecular pathways related to the risk model identified through KEGG and GSEA enrichment analysis. (**A**) Pathway enrichment analysis using KEGG databases. (**B**) GSEA analysis revealing enriched pathways in the high-risk group and low-risk group.
