# Supplementary material for: Identification and validation of anoikis-related lncRNAs for prognostic significance and immune microenvironment characterization in ovarian cancer
Source: Aging (Albany NY). 2024 Jan 15;16(2):1463–83. doi: 10.18632/aging.205439 (PMC10866438; doi:10.18632/aging.205439)
Supplement: Supplementary Tables [file aging-16-205439-s002.pdf]

SUPPLEMENTARY TABLES

Supplementary Table 1. Clinicopathological characteristics and risk group of 5 arlncRNAs in 421 patients with ovarian cancer in TCGA.

	High-risk group	Low-risk group
	**(*N* = 211)**	**(*N* = 210)**
**Age**		
≤60	118 (55.9%)	114 (54.3%)
>60	93 (44.1%)	96 (45.7%)
**Stage**		
I/II	13 (6.2%)	12 (5.7%)
III	164 (77.7%)	165 (78.6%)
IV	32 (15.2%)	32 (15.2%)
Missing	2 (0.9%)	1 (0.5%)
**Status**		
Survive	73 (34.6%)	86 (41.0%)
Dead	138 (65.4%)	124 (59.0%)

Supplementary Table 2. The specific sequences of primers required for qRT-PCR and siRNA.

Target	Sequence
LINC01094 RT primer	F: TGTAAAACGACGGCCAGT<br>R: CAGGAAACAGCTATGACC
AC106801.1 RT primer	F: AATAAGCCTAACCATTACCATAG<br>R: TTGTTGAAGCGTGGAGATT
PRR34-AS1 RT primer	F: CCGCGATTTGGCGTTAACTT<br>R: TCCAAAGATGGCCTCGGTTC
SPAG5-AS1 RT primer	F: AACTTTGCTGAAGAGGCGGA<br>R: TATGGCAGGAAGGACATTGGG
CACNA1G-AS1 RT primer	F: TGTGCTTCACCATGCTCCAT<br>R: TTAGTGCTC CGGCCAACAA
siPRR34-AS1	UAAGUUAACGCCAAAUCGCGG (dT)(dT)
siSPAG5-AS1	GGAGGUUGUUCAUGGUAAA (dT)(dT)
